# Complications of Endoscopic Skull Base Surgery for Sellar and Parasellar Tumors in Pediatric Population; Neurosurgical Perspectives

**DOI:** 10.3389/fonc.2022.769576

**Published:** 2022-05-27

**Authors:** Jeyul Yang, Yong Hwy Kim, Ji Hoon Phi, Seung-Ki Kim, Kyu-Chang Wang

**Affiliations:** ^1^ Neuro-Oncology Clinic, National Cancer Center, Goyang, South Korea; ^2^ Department of Neurosurgery, Seoul National University Hospital, Seoul National University College of Medicine, Seoul, South Korea; ^3^ Division of Pediatric Neurosurgery, Seoul National University Children’s Hospital, Seoul, South Korea

**Keywords:** endoscopic endonasal surgery, children, complication, sellar tumors, parasellar tumors, skull base

## Abstract

**Background:**

Advances in surgical techniques based on in-depth anatomical knowledge of the skull base have broadened the indications for endoscopic skull base surgery (ESS) with the advantage of wide and direct surgical exposure while minimizing invasiveness. However, the low incidence of the indicated diseases and narrow surgical corridors in children have limited the popularization of ESS. In addition, surgical complications and preventive interventions are not yet well known. Therefore, we retrospectively investigated the complications and prevention methods of ESS in children with a comprehensive review.

**Methods:**

We retrospectively analyzed the medical records of pediatric patients who underwent ESS for sellar and parasellar tumors at Seoul National University Children’s Hospital from July 2010 to December 2020. Visual and endocrine status, extent of resection, complications, and recurrences were investigated depending on the pathology of the tumor. In addition, a comprehensive literature review regarding the complications of pediatric ESS was performed.

**Results:**

A total of 98 patients were enrolled. The median age of the patients was 12 years, and 52 patients were male. Preoperative visual disturbance was found in 53 patients, anterior pituitary function deficit in 69, and diabetes insipidus in 32. Gross total resection was attempted in 67 patients and achieved in 62 (93%). Biopsy and cyst fenestration were the goals of surgery in 26 patients, and all were achieved as planned. Regarding outcomes, visual disturbance worsened in two patients (2%), endocrine status was aggravated in 34 (35%) patients, and new-onset diabetes insipidus occurred in 27 (41%) patients. The overall surgical complication rate (other than aggravation of visual or endocrine status) was 17%. Postoperative meningitis (12%) was the most common complication, followed by cerebrospinal fluid leakage (2%), vasospasm, hemorrhage and infarction. By pathological diagnosis, craniopharyngioma had the highest complication rate of 29%. All but one patient with postoperative hemorrhage showed no permanent deficits.

**Conclusion:**

ESS in children is feasible and relatively safe. More attention and different postoperative management protocols are required in children to avoid complications, especially in craniopharyngiomas. However, the complications can be mostly managed conservatively without permanent neurologic deficits.

## Introduction

The transnasal approach to skull base and intracranial structures has developed throughout human history since the ancient Egyptian period. Radiological and endoscopic studies in mummified skulls showed deliberate destruction of the cribriform plate and sphenoid sinuses (1, 2). After adoption of the transsphenoidal approach in modern surgery by Harvey Cushing and Oskar Hirsch, research on a comprehensive understanding of skull base anatomy and development of adequate surgical instruments and techniques have been continuously conducted to overcome abysmal lesions of the human cranium (3).

The endoscope provides wide and angled views compared to those of the microscope, and these features have brought about its implementation in various neurosurgical fields, especially the sellar and parasellar regions, which are the most central parts of the cranium (4). Procedures using an endoscope can be performed in a small working space without a risk of neurovascular damage. In contrast, a long learning curve and less intuitive surgical environments are the major hurdles of endoscopic skull base surgery (ESS) (5). In addition, the narrower nasal cavity and developing sphenoid sinus in children raise concerns about surgical freedom.

In contrast to abundant studies in adults, little is known about ESS in children due to the low incidence of lesions for which ESS is indicated (6). Sellar and parasellar regions, the most indicated surgical fields, are surrounded by critical structures such as the circle of Willis and the hypothalamus, and damage to such structures could be catastrophic. Recognizing types of complications and establishing prevention protocols for complications are the cornerstone of the safety of all surgical procedures. In this study, we retrospectively analyzed the results of ESS in a single institution to elucidate the types, causes and precautions against complications after ESS in children.

## Materials and Methods

A retrospective analysis of patients under 19 years old who underwent ESS for sellar and parasellar tumors at Seoul National University Children’s Hospital from July 2010 to December 2020 was conducted. Medical records, including visual and endocrine status, extent of resection, complications, and recurrences, were investigated depending on the pathology of the tumor.

The major determining factors for the surgical approach were the extent of the tumor invasion and the relative location to the critical neurovascular structures. ESS was not the procedure of choice if the tumors had frontal extension with a cortical cuff or far lateral extension to the internal carotid artery (ICA) bifurcation. Posterior fossa involvement of the tumor was not considered a determining factor because the combined transclivus approach provides a direct approach to the anterior area of the brainstem. In our early experiences, we preferred the transcranial approach for patients under 6 years old due to concerns about a narrow surgical corridor, poor cooperation with postoperative nasal care and cerebrospinal fluid (CSF) diversion such as lumbar drainage in the ESS. Nevertheless, with the accumulation of experience, we lowered the age limitation to 4 years. The pituitary stalk was saved during tumor resection as much as possible, except for cases where tumor invasion into the stalk was evident.

The extent of tumor resection was planned regarding the preoperative imaging studies and surgical role for suspected individual tumor pathology. We always compared the risk of intraoperative neurovascular damage against the benefits of total resection, and the tumor was resected as much as was safely achievable.

The extent of resection was determined according to the postoperative MRI that was obtained within 48 hours after the tumor resection. Gross total resection (GTR) was defined as no evidence of residual tumor, whereas subtotal resection (STR) was defined when a remnant tumor was identified. Biopsy was defined as obtaining a tissue specimen for diagnostic purposes, and fenestration was defined when a connection of the tumor cyst to the extratumoral space was intentionally made. In addition, the achievement rate of the preoperative goal of surgical resection was calculated.

Ophthalmologic evaluation was performed prior to surgery and at six months postoperatively. Visual acuity and visual field were evaluated with Goldmann or Humphrey perimetry, and pre- and postoperative visual statuses were compared. Preoperative visual function was dichotomized as normal or disturbed, whereas postoperative visual function was categorized as improved when perimetry showed improved results, no change, or aggravation.

Pre- and postoperative endocrine statuses were evaluated as reported in our previous study (7). For the evaluation of anterior pituitary function, serum was sampled in the early morning, and the levels of adrenocorticotropic hormone, cortisol, estradiol, follicle-stimulating hormone, growth hormone, insulin-like growth factor-1 (IGF-1), luteinizing hormone, prolactin, testosterone, thyroid-stimulating hormone (TSH), total T4 and free T4 were measured. Preoperative endocrine status was categorized as a partial deficiency when at least one of the anterior pituitary axes showed a deficiency but not all, and panhypopituitarism, when all anterior hormones were decreased. Postoperative hormone changes were checked at one and three months after surgery. Postoperative endocrine status was categorized into improved, no change or aggravated. For cases with low cortisol levels or symptoms of hypocortisolism, a rapid adrenocorticotropic hormone stimulation test was performed. A diagnosis of diabetes insipidus (DI) was made when patients showed polyuria and polydipsia with serum and urine electrolyte imbalances. If the patient showed deficiency in anterior pituitary hormones, DI was diagnosed after appropriate hormonal replacement. Preoperative status and postoperative changes in DI were analyzed.

Complications included the following: CSF leakage, meningitis, vasospasm (without infarction), hemorrhage, and infarction. All complications were proven by laboratory examinations or imaging findings. Complications were categorized as early and late complications. Early complication was defined as a complication that occurred within 14 days. Complications that occurred after 14 days were defined as a late. The criterion was determined as a median value. In addition, complications according to learning curve was measured.

SPSS Statistics version 25 (SPSS Inc., Chicago, IL, USA) was used for statistical analyses. Analyses were performed to demonstrate preoperative manifestations, postoperative outcomes and complications according to tumor pathology. The chi-square test or Fisher’s exact test was used to compare the categorical variables. Student’s t-test or the Wilcoxon rank-sum was used for continuous variables. For comparisons among multiple groups, the Kruskal–Wallis test or Mann–Whitney U test was used. Analyses were two-sided, and a p value <.05 was considered significant. The present study was approved by the institutional review board (IRB) at Seoul National University (SNUH IRB no. 2101-160-1191). Since this research is a retrospective study, informed patient consent was waived by the IRB.

## Results

### Demographics

A total of 98 patients were enrolled in this study. Overall, 52 patients (53%) were male, and 46 patients (47%) were female. The median age at surgery was 12 years, and the median follow-up duration was 59 months (**Table 1**). Craniopharyngioma was the most common (49%), followed by Rathke’s cleft cyst (RCC, 19%) and pituitary adenoma (16%). The others were germinoma, chordoma, Langerhans cell histiocytosis (LCH), hypothalamic hamartoma and optic pathway glioma. The median age was the youngest in chordoma (7 years, range 6–10 years) and the oldest in pituitary adenoma (14 years, range 10–17 years), reflecting the rarity of sellar and parasellar tumors at young ages.

**Table 1:** Overall demographics and preoperative clinical manifestations.

Variables	Overall (n = 98)	Craniopharyngioma (n = 48)	RCC (n = 19)	Pituitary adenoma (n = 16)	Germinoma (n = 8)	Chordoma (n = 3)	LCH (n = 2)	Others (n = 2)	P-value
Median age (yrs, range)	12 (4-18)	12 (4-18)	10 (6-17)	14 (10-17)	12 (9-15)	7 (6-10)	10 (10-11)	11 (6-16)	.056
Sex (%)									.415
Male	52 (53)	30 (62)	9 (47)	6 (38)	2 (25)	2 (67)	1 (50)	2 (100)	
Female	46 (47)	18 (38)	10 (53)	10 (62)	6 (75)	1 (33)	1 (50)	–	
Median follow-up duration (months)	59	58	41	68	74	100	50	93	.633
Hydrocephalus* (%)	11 (11)	10 (21)	–	–	1 (9)	–	–	–	.041
Visual status† (%)									.164
Normal	45 (46)	19 (40)	8 (42)	9 (56)	5 (62)	2 (67)	1 (50)	1 (50)	
Disturbed	53 (54)	29 (60)	11 (58)	7 (44)	3 (38)	1 (33)	1 (50)	1 (50)	
Anterior pituitary function‡ (%)									.023
Normal	29 (30)	7 (15)	7 (36)	10 (62)	3 (38)	2 (67)	–	–	
Partial deficiency	28 (29)	15 (31)	6 (32)	–	3 (38)	1 (33)	2 (100)	1 (50)	
Panhypopituitarism	41 (41)	26 (54)	6 (32)	6 (38)	2 (24)	–	–	1 (50)	
Diabetes insipidus§ (%)	32 (33)	20 (42)	5 (26)	–	7 (88)	–	–	–	.153

*Includes headache, seizure, altered consciousness.

†Includes blurred vision, visual field defect.

‡Includes amenorrhea, galactorrhea, sudden weight gain, short stature, morphologic change.

§Includes polydipsia, polyuria.

RCC, Rathke's cleft cyst; LCH, Langerhans cell histiocytosis.

### Preoperative Neurologic and Endocrine Presentations by Pathologies

Eleven patients suffered from obstructive hydrocephalus, mostly craniopharyngiomas (10 patients), and one suffered from a large suprasellar germinoma (p=0.041). Visual disturbances were present in 60% of patients with craniopharyngioma, 58% of RCCs, 44% of pituitary adenomas, and 38% of germinomas. Anterior pituitary hormone deficiency was found to be significant in craniopharyngioma (85%), followed by RCC (64%), germinoma (62%) and pituitary adenoma (38%) (p=0.023). The management of DI was required in 88% of patients with germinoma, 42% with craniopharyngioma, 26% with RCC and none with other pathologies.

### Goal of Surgical Resection and the Actual Extent of Resection

GTR was the goal for tumors where complete resection was the treatment of choice, such as craniopharyngioma, pituitary adenoma, and chordoma (**Table 2**). The achievement rate of GTR was 94% for craniopharyngioma, 88% for pituitary adenoma, and 100% for chordoma. For all RCCs, cyst fenestration was the goal, and it was achieved in all cases.

**Table 2:** Goal of surgical resection and extent of resection.

Variables (%)		Overall (n = 98)	Craniopharyngioma (n = 48)	RCC (n = 19)	Pituitary adenoma (n = 16)	Germinoma (n = 8)	Chordoma (n = 3)	LCH (n = 2)	Others (n = 2)
Goal of surgical resection (achieved/ attempted)	GTR	62/67 (93)	45/48 (94)	–	14/16 (88)	–	3/3 (100)	–	–
	STR	4/5 (80)	–	3/3 (100)	–	–	–	0/1 (0)	1/1 (100)
	Biopsy	10/10 (100)	–	–	–	8/8 (100)	–	1/1 (100)	1/1 (100)
	Fenestration	16/16 (100)	–	16/16 (100)	–	–	–	–	–
Extent of resection (total)	GTR	62 (64)	45 (94)	–	14 (88)	–	3 (100)	–	–
	STR	9 (9)	3 (6)	3 (16)	2 (12)	–	–	–	1 (50)
	Biopsy	11 (11)	–	–	–	8 (100)	–	2 (100)	1 (50)
	Fenestration	16 (16)	–	16 (84)	–	–	–	–	–

RCC, Rathke’s cleft cyst; LCH, Langerhans cell histiocytosis; GTR, Gross total resection; STR, Subtotal resection.

Three craniopharyngioma patients (6%) and two pituitary adenoma patients (12%) received STR. Biopsy was the goal for germinoma and LCH patients, and was achieved in all patients. STR was planned and performed for hypothalamic hamartoma and biopsy was done for optic glioma for diagnostic purpose.

### Postoperative Neurologic and Endocrine Outcomes by Pathology

Hydrocephalus was resolved in all patients with craniopharyngioma by tumor resection without additional CSF diversion procedures. Similarly, hydrocephalus was improved in a germinoma patient with immediate chemotherapy and radiation treatment after pathological diagnosis through ESS.

Overall, 57 patients (58%) had no change in visual function, whereas 39 patients (74%) of 53 with preoperative visual disturbance showed improvement (**Table 3**). Regarding pathologies, RCC patients showed improvement in 10 out of 11 patients (91%) with preoperative visual disturbance, whereas 72% (21/29) of craniopharyngiomas, 57% (4/7) of pituitary adenomas, 67% (2/3) of germinomas, and 100% (1/1) of chordomas and LCHs showed improvement. The two patients who had aggravated vision were recurrent craniopharyngioma patients, and one of the two patients had preoperative normal visual function. Most patients without preoperative visual disturbance (44/45 patients, 98%) remained unchanged after surgery.

**Table 3:** Postoperative neuroendocrine outcomes and complications.

Variables (%)	Overall (n = 98)	Craniopharyngioma (n = 48)	RCC (n = 19)	Pituitary adenoma (n = 16)	Germinoma (n = 8)	Chordoma (n = 3)	LCH (n = 2)	Others (n = 2)	P-value
Visual outcome									.279
Improved*	39/53 (74)	21/29 (72)	10/11 (91)	4/7 (57)	2/3 (67)	1/1 (100)	1/1 (100)	0/1 (0)	
No change	57 (58)	25(52)	9 (47)	12 (75)	6 (75)	2 (67)	1 (50)	2 (100)	
Aggravated	2 (2)	2 (4)	–		–	–	–	–	
Anterior pituitary function									.047
Improved†	12/69 (17)	4/41 (10)	1/12 (8)	4/6 (67)	0/5 (0)	1/1 (100)	2/2 (100)	0/2 (0)	
No change	52 (53)	23 (48)	12 (63)	11 (69)	3 (38)	2 (67)	–	1 (50)	
Aggravated	34 (35)	21 (44)	6 (32)	1 (6)	5 (62)	–	–	1 (50)	
Diabetes insipidus									.018
Improved‡	4/32 (13)	1/20 (5)	1/5 (20)	–	2/7 (29)	–	–	–	
New onset§	27/66 (41)	24/28 (86)	1/14 (7)	1/16 (6)	1/1 (100)	0/3 (0)	0/2 (0)	0/2 (0)	
Complications									.007
CSF leak	2 (2)	–	2 (11)	–	–	–	–	–	
meningitis	12 (12)	11 (23)	–	–	1 (13)	–	–	–	
Vasospasm	1 (1)	1 (2)	–	–	–	–	–	–	
Hemorrhage	1 (1)	1 (2)	–	–	–	–	–	–	
Infarction	1 (1)	1 (2)	–	–	–	–	–	–	
Recurrence	18 (18)	10 (21)	2 (11)	3 (19)	1 (13)	1 (33)	–	1 (50)	.860

*Percentage based on patients with only preoperative visual disturbance.

†Percentage based on patients with only preoperative anterior pituitary insufficiency.

‡Percentage based on patients with only preoperative diabetes insipidus.

§Percentage based on patients without preoperative diabetes insipidus.

RCC, Rathke's cleft cyst; LCH, Langerhans cell histiocytosis.

Among the patients with total resection as the primary goal of the treatment, only one chordoma patient who had preoperative pituitary insufficiency was normalized after surgery. Two-thirds of the patients [65% (64/98)] improved or had no change in their pituitary function. By pathologic entity, 67% (4/6) of pituitary adenoma patients, 10% (4/41) of craniopharyngioma patients and 8% (1/12) of RCC patients who had preoperative pituitary insufficiency had at least partial improvement after surgery (p=0.047). Aggravation of anterior pituitary function was found in 44% of craniopharyngiomas (21/48) and 32% of RCCs (6/19). Notably, 62% (5/8) of germinoma patients in whom only biopsy was performed showed postoperative endocrine aggravation.

DI improved after tumor resection in 13% (4/32) of the patients, including one patient each with craniopharyngioma (5%) and RCC (20%). In contrast, new DI occurred in 41% (27/66) of the patients after surgery: 86% (24/28) of craniopharyngiomas, 7% (1/14) of RCCs and 6% (1/16) of pituitary adenomas.

### Surgical Complications (Excluding Visual or Endocrine Aggravations)

The overall surgical complication rate of pediatric ESS was 17%, and meningitis was the most common (12/98, 12%) (**Table 3**). Most of the complications occurred in craniopharyngioma patients (p=0.007). All postoperative hemorrhages and infarctions occurred in craniopharyngioma patients. Meanwhile, CSF leakage occurred in 2 patients (2%), and all of them occurred in RCC patients (2/19, 11%). Most of the complication cases fully recovered except for one case with a mild neurologic deficit and one case with a severe deficit.

Early complications were hemorrhage, vasospasm, infarction and meningitis. Cases of early complications are discussed as an illustrative cases. meningitis occurred median 6 days after surgery (range, 3-27 days, mean, 9 days). The two cases of CSF leakage occurred at 17, 19 days after surgery. (**Table 4**) Regarding learning curve, no neurological morbidity occurred after 3 years of ESS initiation of a single neurosurgeon.

**Table 4:** Chronology of postoperative complications.

Early (≤14 days)	Late (>14 days)
Hemorrhage	CSF leakage
Infarction	
Vasospasm	
Meningitis	

The overall recurrence rate was 18% (18 patients). Except for one patient with optic pathway glioma in whom simple decompression with STR was the goal of surgical resection, chordoma had the highest recurrence rate of 33% (1/3), followed by craniopharyngioma (10/48, 21%) and pituitary adenoma (3/16, 19%).

## Illustrative Cases

### Case 1

**Figure 1:**
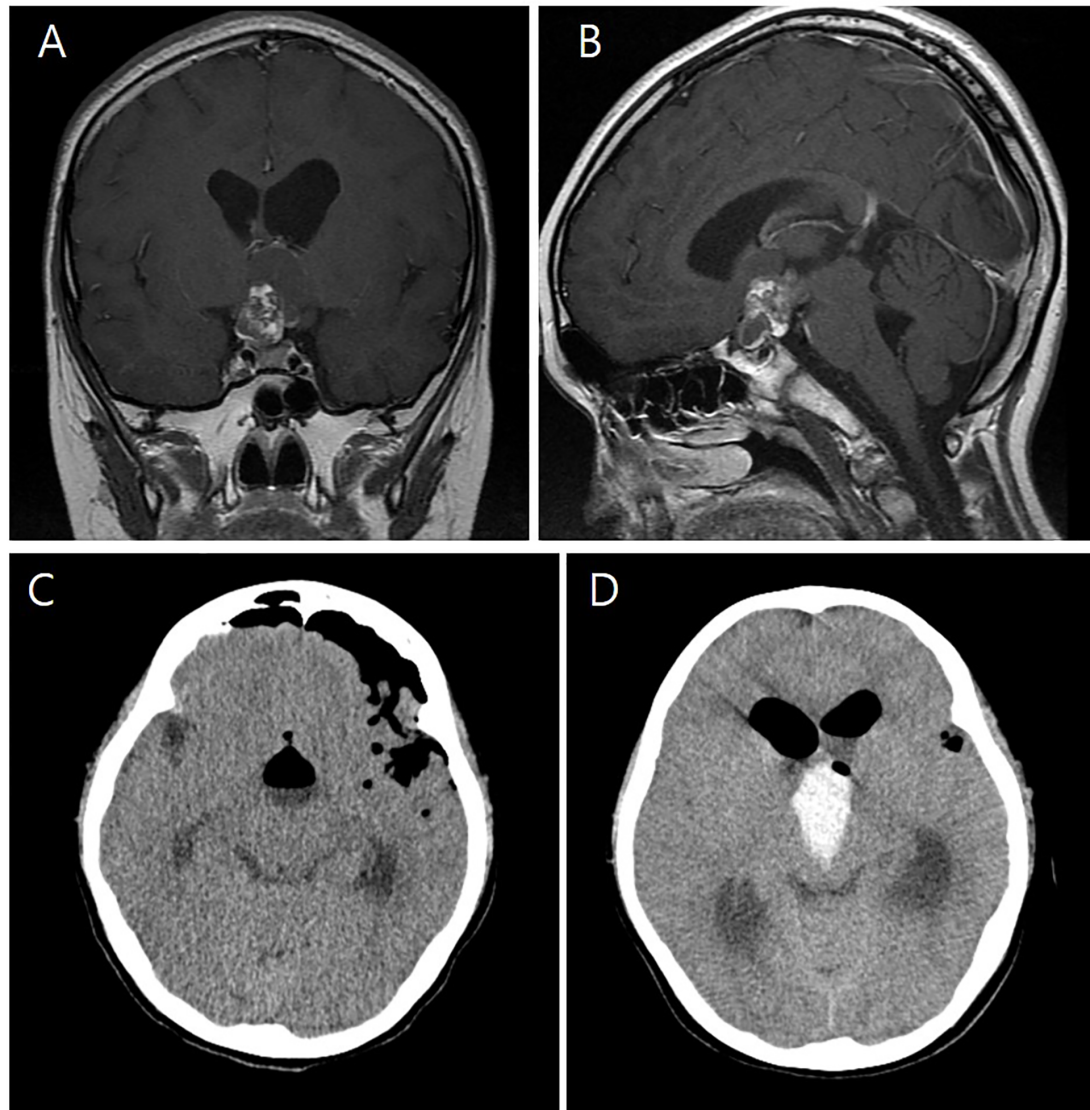
Preoperative T-1 enhanced coronal **(A)** and sagittal **(B)** images of an eight-year-old girl showing a 3.4 cm × 2.5 cm × 1.7 cm craniopharyngioma with cystic and solid components and calcification in the suprasellar area. Immediate postoperative brain CT shows no abnormal findings **(C)**. However, on the first postoperative day, intraventricular hemorrhage was found in the third ventricle **(D)**.

An eight-year-old female presented with headache and vomiting one week prior. MRI revealed a 3.4 cm×2.5 cm×1.7 cm cystic and solid tumor with calcification causing obstructive hydrocephalus. (**Figure 1**) The patient underwent ESS for tumor resection. The surgery was uneventful, and immediate postoperative CT also showed no visible mass or hemorrhage. She woke up well in the pediatric intensive care unit; however, she showed poor cooperation, such as removing the endotracheal tube by herself. She complained of discomfort about the nasal packing installed to prevent epistaxis and tried to blow her nose intensely. Nine hours after surgery, she vomited several times and showed a rigid posture. Brain CT was immediately performed, and intraventricular hemorrhage was found in the third ventricle. Emergency extraventricular drainage was performed to control her intracranial pressure, and then craniectomy and frontal lobectomy were performed. Unfortunately, however, she was unable to recover, and severe permanent neurocognitive dysfunction remained. After this case, we changed the postoperative care protocol to keep the patients in light sedation, even if no hemorrhage was seen on immediate postoperative CT, except those for whom the goal of surgical resection was cyst fenestration, which carries a low risk of hemorrhage.

### Case 2

**Figure 2:**
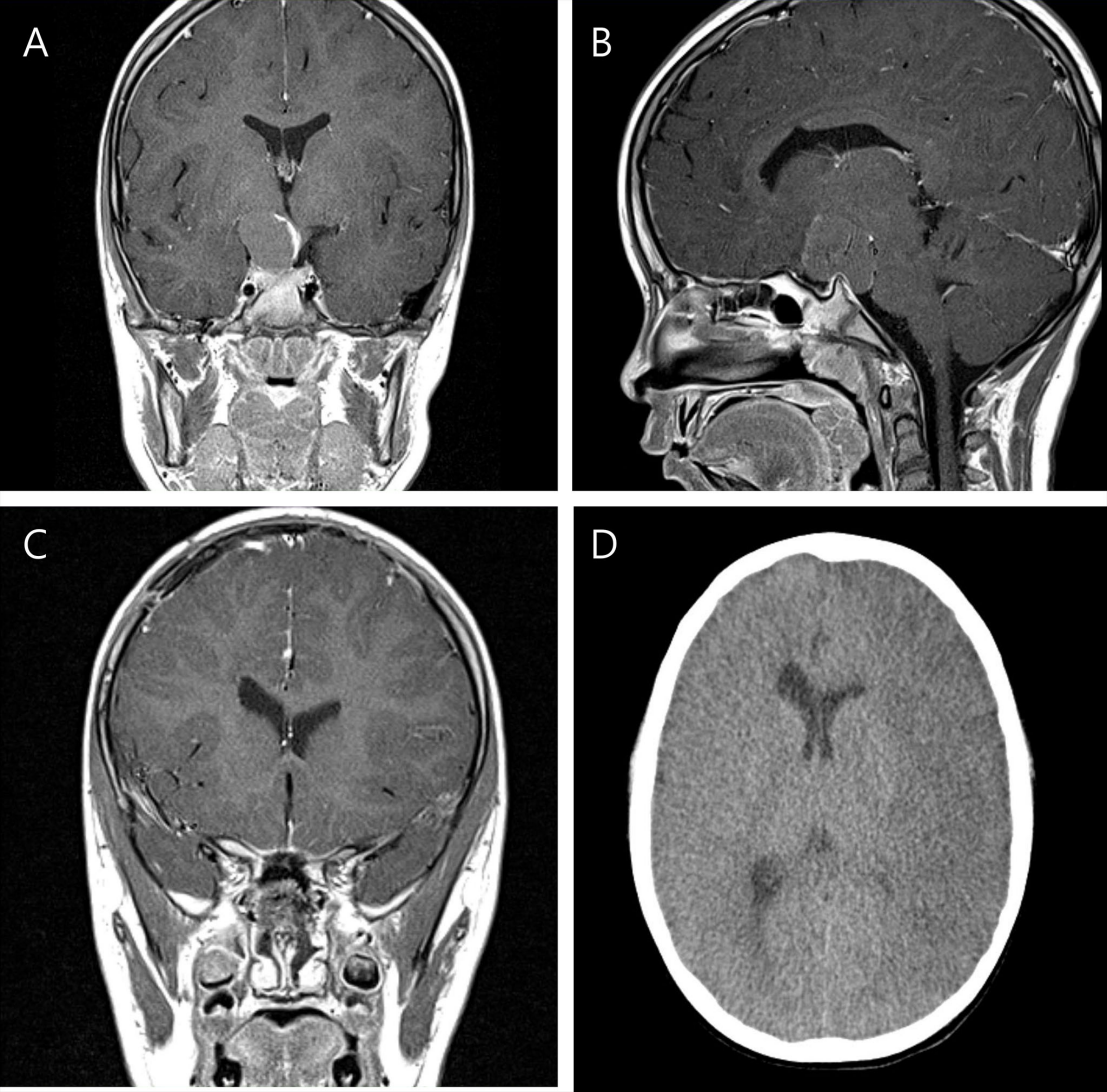
Preoperative T-1 enhanced coronal **(A)** and sagittal **(B)** images of a six-year-old boy with craniopharyngioma. Postoperative MRI taken within 48 hours shows successful removal of the tumor without any abnormal finding **(C)**. However on postoperative day 6 after rapid fluctuation of his serum sodium level, low attenuation along the MCA territory was found **(D)**, suggestive of cerebral infarction.

A six-year-old male underwent ESS for recurrent craniopharyngioma. (**Figure 2**) The patient had a history of craniotomy with tumor resection four years ago and gamma knife surgery for recurrence one year ago at another institution. In addition, he had received hormone replacement therapy for panhypopituitarism. ESS was performed uneventfully, and the patient was on routine postoperative care. No remarkable finding was observed on his postoperative images, including MRI. However, his serum sodium level started to fluctuate in the range of 123 mmol/L to 152 mmol/L from postoperative day (POD) 2. As a result, his level of consciousness deteriorated to a stupor, and the left pupil was dilated. Brain CT showed uncal herniation without definite hemorrhage or infarction. His serum sodium level ranged from 144 mmol/L to 153 mmol/L on POD 3. His pupil size was normalized, and the uncal herniation showed improvement on CT, although he could not obey commands. His level of serum sodium slowly decreased from 150 mmol/L to 136 for the following two days, and he became alert again. However, on POD 6, his sodium level decreased sharply from 133 mmol/L to 125 mmol/L for five hours, and his physical activity also decreased significantly. CT showed left middle cerebral artery territory infarction. After intensive care for electrolyte imbalance, the patient recovered with right hemiparesis. Fortunately, his right hemiparesis was nearly normalized after six years of rehabilitation.

### Case 3

**Figure 3:**
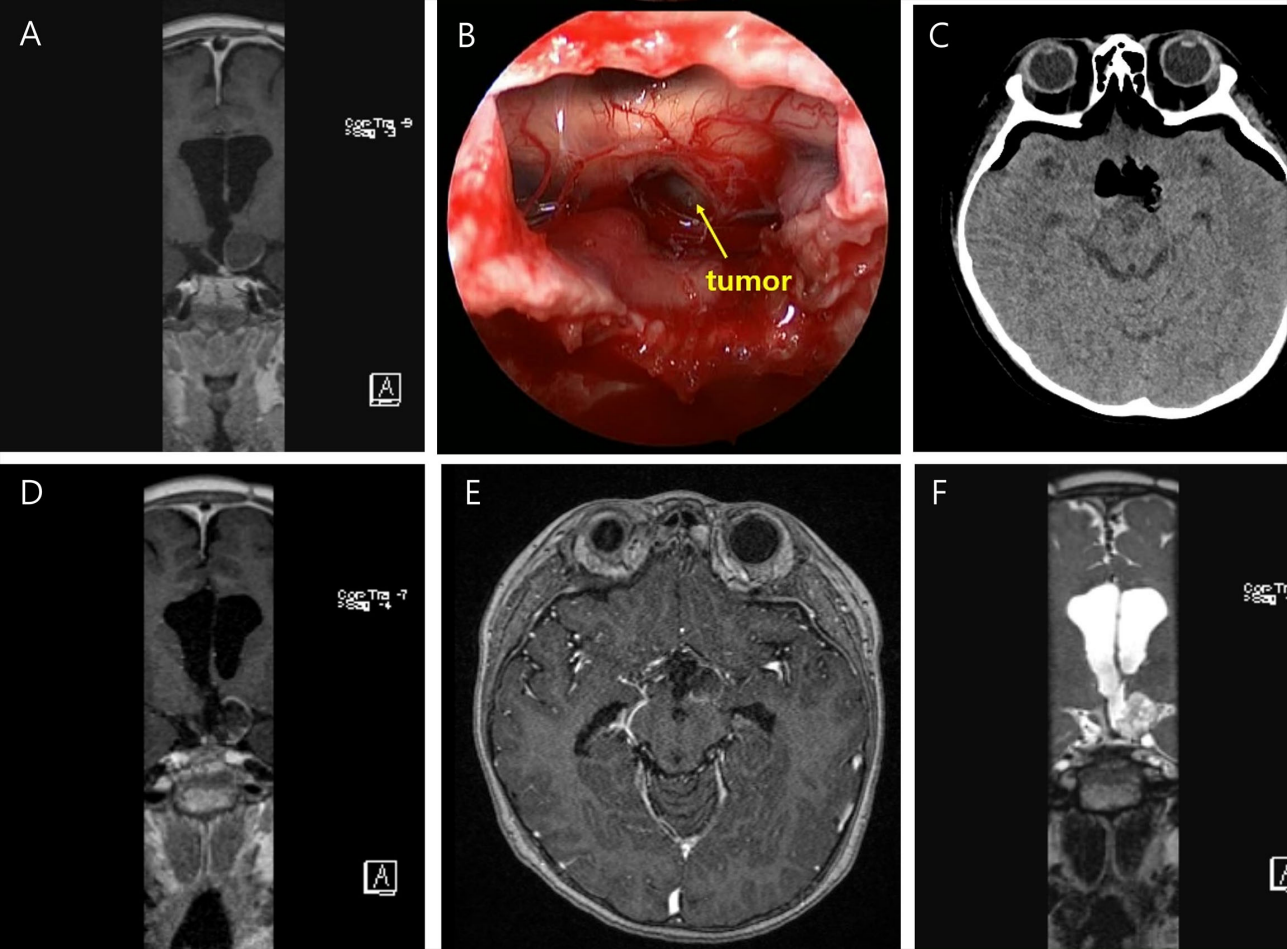
Preoperative T-1 enhanced coronal image of a six year-old male who underwent ESS for recurred craniopharyngioma **(A)**. The tumor is visualized under optic chiasm **(B)**. Immediate postoperative brain CT shows no abnormal finding **(C)**. Postoperative T-1 enhanced coronal **(D)**, axial **(E)**, and T-2 coronal **(F)** MRI taken within 48 hours after surgery showed no abnormal signal intensities.

A six-year-old male underwent ESS for recurrent craniopharyngioma. (**Figure 3**) He underwent the transcranial approach one year ago. After dural opening, the right superior hypophyseal artery was well visualized. In contrast, the left superior hypophyseal artery was covered by scar-like fibrous tissue. The tumor was located medial to the optic tract and inferior to the hypothalamus and adhered severely to them. Liliequist’s membrane was also thick with fibrotic changes. The tumor was removed by dissecting along the gliotic plane, and no unexpected event occurred. An MRI taken within 48 hours after surgery showed no abnormal signal intensities. Nevertheless, on POD 2, the patient complained of visual disturbance. Ophthalmologic evaluation found medial hemianopsia of the left eye. Regarding the onset time of the symptoms and the defective visual field, vasospasm was suspected. Steroid and nimodipine therapy was administered. The patient is currently on routine follow-up, and his subjective visual field has slightly improved. No definite sign of infarction was visible on MRI.

## Discussion

ESS has shifted the surgical paradigm of skull base tumors from unreachable, technically challenging and time-consuming to a better visualized, minimally invasive and less time-consuming procedure (8). Technical advancements and the accumulation of experiences have widened the indications for ESS in children (9). Considering skull base development, sphenoid pneumatization and intercarotid distance, ESS is considered acceptable in most pediatric patients over the age of three (10–12). Our youngest experience was four years old.

### Goal of Surgical Resection

Our goal of surgical resection were mostly achieved, except for the three cases with craniopharyngiomas and the two cases with pituitary adenomas. All but one case who were not able to achieve the surgical goal were recurrent cases who had undergone previous surgery including radiosurgery, which is a risky process with higher chance of morbidity. Once on track, ESS in children appears to be an effective tool in dealing with skull base tumors in selected cases, which is in line with previous report by Deopujari et al. (13).

### Ophthalmologic Outcome

ESS for sellar and parasellar regions carries the risk of ophthalmologic complications, such as visual disturbance or diplopia, due to the intimate nature of the cranial nerves and supplying vessels. In contrast to the high incidence of pituitary adenoma in adults, which has less influence on the suprasellar area and optic nerves, craniopharyngiomas are common in children and are more likely to affect such regions. The risk of postoperative visual deterioration with pituitary adenoma is lower than that of craniopharyngioma because pituitary adenomas mostly arise from the pituitary gland and tend to grow beneath the relatively thick diaphragma sellae, which serves as an anatomical barrier to optic nerves during surgical procedures. Likewise, RCCs share these anatomical features and are relatively safe from visual deterioration after surgery, as shown in our results. In contrast, craniopharyngiomas, except for the subdiaphragmatic type, usually originate from the pituitary stalk. The arachnoid membrane wrapping the pituitary stalk and pia of the tuber cinereum serves as the dissecting plane from the superior hypophyseal artery, optic nerves and tract (14, 15). Therefore, it is essential to pay careful attention when opening the dura during ESS to protect the superior hypophyseal artery and optic nerve, especially in cases where the optic nerves and chiasm are displaced antero-inferiorly due to hydrocephalus. After opening the dura, it is our tip to dissect the outer arachnoid membrane of the chiasmatic cistern from the diaphragma sellae. By doing so, the superior hypophyseal artery is protected from direct manipulation. In addition, vasospasm of the artery is prevented because the superior hypophyseal artery is covered by the outer arachnoid membrane which serves as the anatomical barrier. If the tumor has to be dissected from the tuber cinereum and optic tract, we try to find the gliotic plane and perform subpial dissection. The visual complications in our cohort only occurred in the recurrent cases, not in the primary craniopharyngioma, reflecting the importance of anatomical features. The lack of an intact arachnoid or pial membrane between the tumor and the optic apparatus and vessels may cause important neurovascular injury.

Extraocular muscle palsy is frequently found in pituitary adenoma, whereas it is rarely found in craniopharyngioma and RCC. This is because craniopharyngioma and RCC do not invade the cavernous sinus. The oculomotor nerves in cisternal spaces are protected by the medial carotid membrane and Liliequist’s membrane, which separate them from the chiasmatic cistern, where craniopharyngiomas are usually located. Therefore, we recommend meticulous dissection of the arachnoid membrane from craniopharyngioma, which rarely carries a risk of oculomotor palsy as shown in our results.

### Endocrine Outcome

Anterior pituitary axis deficiency develops in the order of somatotropin, gonadotropin, corticotropin and then TSH due to the different vulnerabilities of the axes. It is impossible to evaluate gonadotropin deficiency in children before puberty, and there is a large variance among individuals in the age of puberty.

For the preoperative assessment of the somatotropin and corticotropin axes, we used the levels of growth hormone, IGH-1, adrenocorticotropic hormone, and total cortisol from the serum sampled at 8 am except in cases of clinically definite hypocortisolism. In our institution, provocation tests for somatotropin and corticotropin are not performed because they may cause hypoglycemic insult in patients with brain lesions. In addition, most of the patients present with neurological deficits such as visual disturbance, and therefore, endocrine deficiency is not a determining factor for the surgery. However, there is a chance of missing subclinical hypocortisolism in our protocol. Hence, provocation tests for somatotropin and corticotropin are included postoperatively for endocrine evaluation because they are essential steps for appropriate long-term hormone replacement therapy in growing children. Our endocrine evaluation can exaggerate the deterioration rate of endocrine status by surgery because of the possible underdiagnosis of preoperative hormonal deficiency, but we emphasize the preoperative safety of the children rather than a precise assessment of individual hormonal status.

Postoperative endocrine outcomes depend on various pre- and intraoperative factors, such as the types of deficient axes, duration of deficiency, anatomical location of the pathology and the degree of surgical resection. We plan different extents of tumor resection based on the pathology: GTR for craniopharyngioma and pituitary adenoma, cyst fenestration and drainage for RCC, and biopsy for germinoma.

Endocrine deterioration rate after ESS for craniopharyngioma seems relatively low in our cohort. However, this is due to the large number of recurrent cases, which already had panhypopituitarism. We have previously reported that endocrinological outcome is determined by the tumor invasion to center of pituitary stalk, rather than preservation of the stalk during surgery (16). Among the primary craniopharyngioma patients whose stalk was preserved, endocrinological function remained only in 43.4% of the patients.

For craniopharynioma, we dissected the tumor from the pituitary stalk and preserved the branches of the superior hypophyseal arteries to the pituitary stalk. However, endocrine aggravation was found in 44% of patients, although it was comparable with other reports with the same surgical policy, even with our higher GTR rate (17, 18). We agree that endocrine outcomes could be improved by adjuvant radiation therapy after STR without manipulation of the hypothalamic-pituitary axis without a difference in recurrence rates from GTR (19). However, because approximately 20% of patients still experience recurrence after GTR or STR followed by irradiation, and the fact that the GTR rate is lower and the complication rate is higher in recurrent cases and that treatment options are limited after radiation led to our GTR policy for craniopharyngioma at the initial surgery despite the risk of hypopituitarism (7).

For pituitary adenoma, we perform extracapsular dissection to avoid mechanical damage to the pituitary gland by blunt curettage. For functioning pituitary adenoma cases with a suspected medial cavernous wall defect, we explore the cavernous sinus. An improvement in only one of our six cases with panhypopituitarism shows the gloomy prognosis of panhypopituitarism in children. Meanwhile, the fact that none of our nine cases with normal pituitary function showed endocrine deterioration, suggests the importance of the preservation of compressed thin pituitary glands with microsurgical dissection techniques.

RCC is a congenital cyst that arises during the development of the pituitary gland, and no consensus on the extent of resection has been reached. Our strategy is partial excision for the pathologic diagnosis and drainage of the cyst contents for symptomatic lesions only. Unfortunately, only one of five patients recovered from preoperative panhypopituitarism, and 32% of patients experienced endocrine deterioration. Four of six deteriorated patients revealed asymptomatic hypocortisolism with an abnormal thyroid axis on the provocation test three months after surgery. We speculate that these might be preoperative hypocortisolism cases that were missed because preoperative provocation tests were not performed.

An interesting finding in our results was the high (62%) endocrine deterioration rate of germinomas. This implicates a risk of hypopituitarism caused by direct surgical injury to the stalk or ischemia from subpial capillary network damage. The cutoff thickness value of the stalk for biopsy was suggested to be in the range of 5.3 mm to 7.0 mm to avoid unnecessary biopsy and related complications (20–22). A biopsy of the pituitary stalk can be delayed until the lesion begins to grow on the follow-up MRI for cases with suspicious nonneoplastic lesions. On the other hand, the high incidence of germ cell tumors in Asians and the low incidence of nonneoplastic lesions of the pituitary stalk in children advocate for biopsy of the pituitary stalk despite the endocrine risk (20, 23).

DI is the symptomatic form of a neurohypophysis disorder, and may occur not only by destruction of the pituitary stalk or neurohypophysis but also by the rapid displacement of the stalk after surgical decompression of the tumor (24). In our study, new-onset DI was frequent in craniopharyngioma and rare in RCC and pituitary adenoma (p=0.018). On the other hand, the most common endocrine presentation of germinoma was DI, owing to damage to the pituitary stalk or neurohypophysis.

### Hydrocephalus

One-fifth of craniopharyngioma patients (10 patients) presented with hydrocephalus at the initial diagnosis. However, none of the patients with hydrocephalus needed additional CSF diversion procedures after tumor resection. The presence of hydrocephalus is reported to have no impact on overall survival, progression-free survival or functional capacity. Therefore, preoperative ventriculoperitoneal (VP) shunting is not recommended.

In contrast, preoperative VP shunting is controversial in germinoma cases. Recently, because of the rapid response of germinoma to chemotherapy and radiation, conservative management for hydrocephalus has been preferred unless there is an aggravation of symptoms and signs by the hydrocephalus.

Deopujari et al. have reported favorable outcomes regarding CSF leak when managed with vascularized flaps in various tumors in children (13). In our series, there were three cases in which ventriculostomy was performed prior to the surgery rather than draining the cyst in the management of hydrocephalus. We agree that if the increased intracranial pressure is life-threatening by hydrocephalus and ESS cannot be performed immediately, ventriculostomy or cyst drainage should be done. In our experience, ventriculostomy causes the ventricle to contract, resulting a narrow third ventricle, which makes the surgical space smaller.

### CSF Leakage and Meningitis

CSF leakage and postoperative meningitis are the Achilles’ heel of ESS. Multilayered reconstruction with pedicled nasoseptal flaps is the most reliable method to prevent CSF leakage, despite the suggestion of various techniques with diverse autologous, allogenic or artificial materials with or without CSF diversion. During our early experience, we reconstructed with fat graft only with lumbar drainage, which resulted in two cases of CSF leakage. Consequently, we revised the reconstruction protocol using buttress-type autologous fascia and pedicled nasoseptal flaps (25). This technique showed excellent results in skull base reconstruction and infection control. However, the additional skin incision in children, the need for a lumbar drain for a long period (5~7 days), difficulties while caring for a lumbar drain in poorly cooperative young patients, and the possibility of adhesion of in-layer fascia to the intracranial structures are problems to be solved. Additionally, the lack of autologous fascia to harvest is another problem for patients with recurrent diseases. To overcome these problems, we suggested new reconstruction techniques for high-flow CSF leakage using multiple onlay artificial materials and pedicled nasoseptal flaps, which did not require postoperative lumbar drainage (26). This technique resolved the shortcomings of previous techniques with favorable CSF leakage prevention. However, aseptic meningitis due to artificial materials occurred in 10 cases, comprising 83% of the postoperative meningitis, although all patients fully recovered without any deficits.

Failure of skull base reconstruction was due to various reasons, such as inappropriate flap size or design, flap necrosis, and increased intracranial pressure. When CSF leakage is highly suspicious, prompt revision surgery to seal the leakage point is the treatment of choice (27).

### Vascular Complications

A survey reported that one-fifth of ESS surgeons experienced various types of ICA injury, and the most common type of ICA injury was perforation or laceration of the cavernous portion (5, 28). Risk factors for ICA injury include a history of prior surgery, radiation and prolonged bromocriptine treatment, tortuous artery due to acromegaly, tumor invasiveness and dehiscent carotid artery (29–31). Hence, recurrent craniopharyngioma with a history of STR followed by various radiation treatments carries the highest risk among the indicated pathologic entities for ESS in children. A midline dural incision is essential to avoid disorientation by the intracranial fibrous scar tissues and to determine whether the arachnoid membrane around the tumor is intact or disrupted.

Microbleeding from small perforators often stops easily by irrigating warm saline, which activates the coagulation cascade (27). Just pinching the bleeding point with an aneurysm clip without compromising blood flow is also an available option if applicable (27). For larger injuries, surgical packing is a commonly used method. Packing with an autograft muscle piece has been shown to be effective (32). The muscle should be crushed and flattened to release calcium for hemostasis (27). Other packing options are hemostatic sponge collagen, muslin gauze patches, or surgical glues (33–36). Occlusion is the last option to be used but should be used without hesitation in cases of massive uncontrolled bleeding that threatens life. Unilateral ICA occlusion with collateral blood flow *via* the anterior or posterior communication artery is associated with a low prevalence of border zone infarcts (37).

Postoperative vasospasm or infarction could be caused by various factors, such as subarachnoid hemorrhage (even when the amount is small), direct surgical manipulation of vessels, or poor general status, such as fluid and electrolyte imbalance, as in our illustrative cases (34). The illustrated case with middle cerebral artery territory infarction had electrolyte imbalance during the immediate postoperative period, which accentuated the vasospasm. The case with superior hypophyseal artery vasospasm resulted in aggravation of vision, probably by concurrently affecting the subchiasmatic perforators.

To prevent vasospasm, we irrigate the intracranial space with normal saline after complete hemostasis and tumor resection and then apply cottonoid pledgets soaked with vasodilating agent over the exposed vessels and the surface of optic nerves and chiasm. Additionally, we keep our patients hydrated sufficiently and administer nimodipine intravenously during the postoperative period.

### Long-Term Effect on the Growing Skull

An adverse effect on midface growth was one of the major concerns in pediatric ESS. A study that divided the age group at the age of 7 demonstrated no difference in sella-nasion distance or the angles between the sella, nasion, and the most concave points of the anterior maxilla or the anterior mandibular synthesis (38). Similarly, no identified difference was found in craniofacial growth between the ESS group and the endoscopic sinus surgery group (39). Therefore, there seems to be no impact of ESS on craniofacial development.

## Conclusion

ESS is an invaluable surgical modality with documented safety for sellar and parasellar tumors in children. Due to the location and growth pattern of the tumor, craniopharyngioma requires more meticulous attention during postoperative care. Surgeons should always be prepared for serious inadvertent complications, although the complications can mostly be managed conservatively without permanent deficits.

## Data Availability Statement

The raw data supporting the conclusions of this article are available from the corresponding authors on a reasonable request.

## Author Contributions

SKK and KCW contributed to the study conception and design. Material preparation, data collection and analysis were performed by JY, YHK, and JHP. The first draft of the manuscript was written by JY and YHK. All authors commented on previous versions of the manuscript. All authors contributed to the article and approved the submitted version.

## Conflict of Interest

The authors declare that the research was conducted in the absence of any commercial or financial relationships that could be construed as a potential conflict of interest.

## Publisher’s Note

All claims expressed in this article are solely those of the authors and do not necessarily represent those of their affiliated organizations, or those of the publisher, the editors and the reviewers. Any product that may be evaluated in this article, or claim that may be made by its manufacturer, is not guaranteed or endorsed by the publisher.
